# Case Report: Long-Term Survival of a Dog With Chronic Lymphocytic Leukemia Treated With Chlorambucil, Prednisolone, and Imatinib

**DOI:** 10.3389/fvets.2021.625527

**Published:** 2022-01-17

**Authors:** Ga-Won Lee, Min-Hee Kang, Jin-Ha Jeon, Doo-Won Song, Woong-Bin Ro, Heyong-Seok Kim, Hee-Myung Park

**Affiliations:** ^1^Laboratory of Veterinary Internal Medicine, College of Veterinary Medicine, Konkuk University, Seoul, South Korea; ^2^Department of Bio-Animal Care, Jangan University, Hwaseong, South Korea

**Keywords:** chlorambucil, chronic lymphocytic leukemia, dog, flow cytometry, imatinib, lymphocytosis

## Abstract

A 7-year-old castrated male Poodle dog presented with chronic progressive lymphocytosis. Hematologic and peripheral blood smear findings included remarkable lymphocytosis with well-differentiated small lymphocytes. Cytology of bone marrow aspirate showed hypercellular integrity with infiltration of small mature lymphocytes, accounting for 45% of all nucleated cells. Flow cytometry of blood and marrow samples revealed neoplastic lymphocytes predominantly expressing the CD21 molecule. B-cell chronic lymphocytic leukemia (CLL) was diagnosed on an immunophenotypic analysis. Administrations of prednisolone and chlorambucil were initiated and the response was unremarkable. Therefore, additional treatment with imatinib was provided, which resolved the hematologic abnormalities associated with CLL. Flow cytometry after ~1 year of treatment showed normalization of the count of lymphocytes positive for CD21 and resolved hematologic lymphocytosis. The dog was followed-up for 2 years, and there were no severe adverse effects. This case indicates that imatinib may be a good option as an adjunctive therapy with prednisolone and chlorambucil treatment for CLL in dogs without treatment response.

## Introduction

Chronic lymphocytic leukemia (CLL) is a lymphoproliferative disease characterized by neoplastic clonal proliferation of small lymphocytes in the bone marrow, which manifests as persistent and marked peripheral lymphocytosis for more than 3 months as previously described (1, 2). The leukemic cells in CLL morphologically resemble mature lymphocytes and migrate throughout the blood and tissues (3). CLL is an indolent disease of older humans (4) and dogs (5), and several studies on dogs with CLL show that the median age of onset is ~10 years, with a range of 1.5–15 years (1, 2, 6). In a previous report (2, 5), CLL has no sex predilection in dogs and occurs less commonly compared to acute lymphoblastic leukemia (ALL) and more commonly than myeloproliferative disorders. The cause of CLL is still unknown, but a strong genetic risk has been identified (1, 5). Clinical signs are non-specific and include lethargy, inappetence, pyrexia, weight loss, vomiting, and mild splenomegaly/lymphadenopathy (3). The demonstration of clonality in the molecular genetic analysis can lead to diagnosis of neoplasia. Immunophenotyping with flow cytometry is useful for obtaining the definitive diagnosis and can identify proliferations of B- and T- lymphocytes (2, 6).

In contrast to ALL and leukemic lymphoma, CLL is defined as a slowly progressing disease characterized by a long subclinical period requiring no treatment (2). Treatment of CLL is required when the animal manifests with clinical signs, is anemic or thrombocytopenic, has lymphadenopathy or hepatosplenomegaly, or has an excessively high lymphocyte count of over 60,000/μL (7). The most effective agents for dogs with CLL are chlorambucil and prednisolone until recently. However, CLL still remains incurable (8, 9). Vincristine can be added to the regimen, and chlorambucil can be replaced with cyclophosphamide (2). Melphalan and prednisolone therapy can be administrated to dogs with CLL, reflecting clinical improvement for 8–210 days as described earlier (3).

In human medicine, chlorambucil or chemotherapy with anti-CD20 antibodies is the standard therapy for CLL (4). Novel targeted therapies for human CLL include B-cell receptor inhibitors, phosphatidylinositol-4,5-bisphosphate 3-kinase inhibitors, anti-CD20 monoclonal antibodies, and Bcl-2 antagonists (4, 10, 11), but until recently, there has been no report of chemoimmunotherapy in canine CLL.

This report first describes the clinical features and long-term outcome of a dog with CLL treated with chlorambucil and prednisolone in combination with imatinib.

## Case Description

### Case Presentation and Diagnostic Investigations

**Figure 1:**
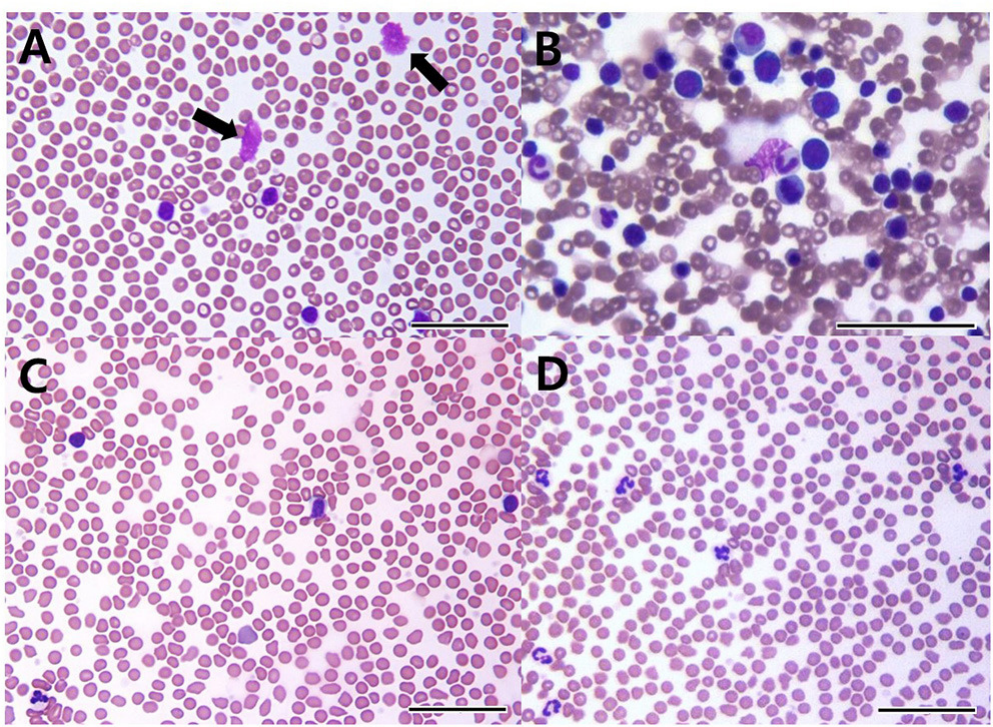
Peripheral blood **(A,C,D)** and bone marrow cytology **(B)** in a dog with B-cell chronic lymphocytic leukemia. On the first visit, cytology shows increased well-differentiated lymphoid cells in peripheral blood **(A)** and the bone marrow **(B)**. Smudge cells (black arrows) are seen in peripheral blood **(A)**. 22 days after initial treatment **(C)**, cytology reveals lymphocytosis despite administration of prednisolone and chlorambucil. After 1 year of multimodality therapy with additional administrations of imatinib, leukocytosis and lymphocytosis are resolved **(D)** [**(A–D)**: bar = 50 μm].

A 7-year-old castrated male Poodle dog was referred for evaluation of persistent lymphocytosis that had lasted for more than 20 months and gradually worsened. Specific infections were ruled out in that canine SNAP 4Dx (IDEXX Laboratories, Westbrook, ME) for heartworm, Lyme, *Ehrlichia canis, Anaplasma phagocytophilum* was all negative before the visit. On physical examination, the dog had hyperthermia (rectal temperature: 39.4°C) and tachypnea. The size of superficial lymph nodes was found to be normal on palpation. Hematologic findings included leukocytosis, lymphocytosis, and neutropenia (Table 1, Day −28). On peripheral blood smear, lymphocytosis with well-differentiated small lymphocytes and neutropenia were found (Figure 1A). Results of serum chemistry were within normal ranges. Abdominal ultrasonography showed hepatosplenomegaly. Chronic inflammation and immune mediated diseases, which could primarily induce persistent lymphocytosis were excluded and lymphoproliferative disease was highly suspected. Therefore, bone marrow aspirate was performed to identify marrow infiltration and to evaluate the cytological types and proportions of hematopoietic cells. Bone marrow aspirate cytology showed hypercellularity and infiltration with small-sized mature lymphocytes (Figure 1B); the percentage of lymphoid cells was 45%. Differential diagnosis for the lymphocytosis of bone marrow include ALL, CLL, and stage V lymphoma. Among them, the dog was diagnosed with CLL based on the results of clinical examination mentioned above.

**Table 1:** Profiles of complete blood counts and serum biochemistry results in a dog with B-cell chronic lymphocytic leukemia.

**Parameters**	D-28^*^	**D0**	**D7**	**D15**	**D22^†^**	**D36**	**D100**	**D113**	**D127**	**D387**	**Reference interval**
WBCs (× 10^9^/L)	36.88	41.2	28.09	44.49	35.46	21.53	30.4	32.32	16.86	15.41	5.05–16.76
Neutrophils (× 10^9^/L)	7.14	15.2	16.72	13.13	13.61	7.75	20	19	9.09	10.23	2.95–11.64
Lymphocytes (× 10^9^/L)	28.63	23.28	9.52	29.04	20.7	11.13	9.17	10.75	6.53	3.2	1.05–5.10
RBCs (× 10^12^/L)	8.25	8.66	7.53	8.7	7.38	6.29	6.39	5.99	7	6.26	5.65–8.87
HCT (%)	51.9	57.4	46.3	53.6	45.4	39.4	39.7	36.7	44.5	39.5	37.3–61.7
Plt (K/μL)	213	235	182	295	352	154	281	160	343	72	148–484
BUN (mg/dL)	25	–	31	23	20	21	13	17	–	19	7–27
CREA (mg/dL)	0.7	–	0.5	0.7	0.7	0.7	0.7	0.8	–	0.7	0.5–1.8
ALT (U/dL)	<10	–	68	147	45	<10	17	11	24	<10	10–100
AST (U/dL)	32	–	44	58	27	23	23	29	–	40	0–50
ALP (U/dL)	36	–	101	573	269	134	101	160	62	165	23–212
Lipase (U/L)	1,711	–	732	–	1,223	1,742	1,432	1,178	–	1,039	200–1,800

* *First visit at the hospital*.

† *The day of additional administration of imatinib to the original chemotherapy. ALP, alkaline phosphatase; ALT, alanine transaminase; AST, aspartate transaminase; BUN, blood urea nitrogen; CREA, creatinine; D, Days after first treatment on which 28 days after the first visit; HCT, hematocrit; Plt, platelet; RBC, red blood cell; WBC, white blood cell*.

**Figure 2:**
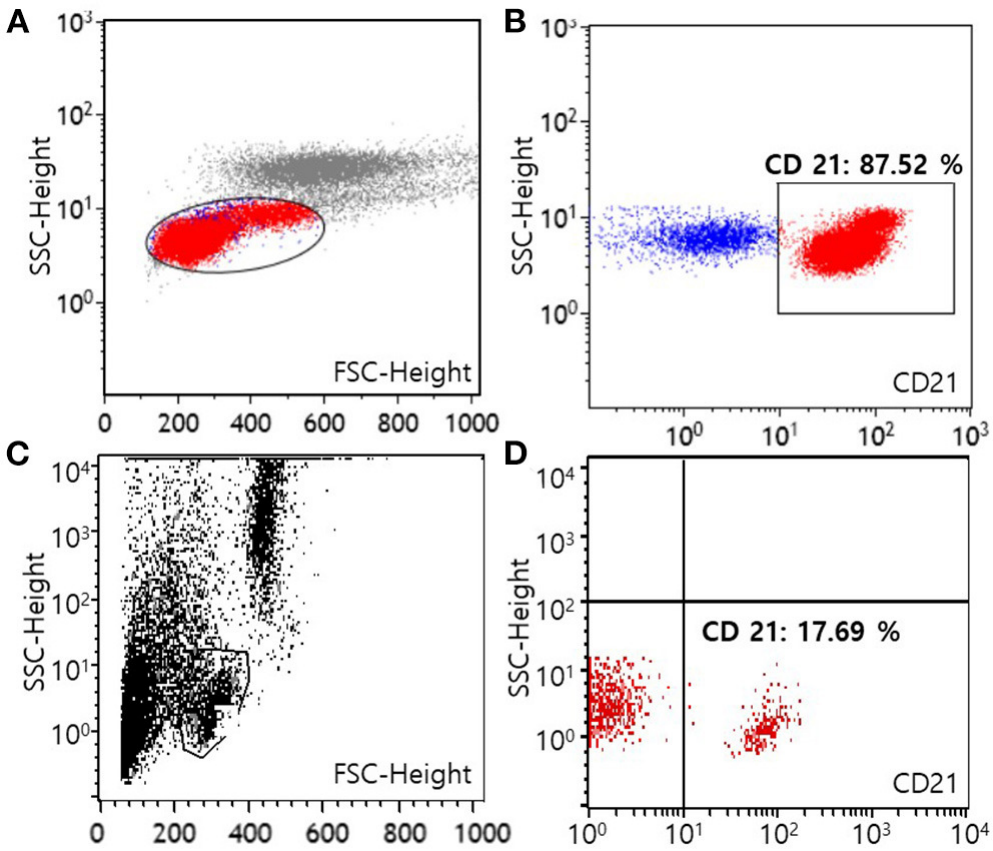
Flow cytometric scatter plots of peripheral blood from a dog with B-cell chronic lymphocytic leukemia at the time of diagnosis **(A,B)** and after 1 year of multimodality therapy **(C,D)**. A template is used to identify lymphocytes **(A,C)**. On the first visit, lymphocytes predominantly express the CD21 molecule (87.52% among lymphocytes), showing a B-cell origin **(B)**. After 1 year of treatment, the number of lymphocytes expressing the CD21 molecule has decreased to normal [17.69% among lymphocytes, **(D)**] (FSC, forward scatter; SSC, side scatter).

For the evaluation of immunophenotype by flow cytometry, both blood and marrow samples were stained with a panel of antisera specific for the T-lymphocyte markers [CD3 (CA17.2A12; Serotec Inc, Raleigh, NC, USA), CD5 (YKIX322.3; Serotec Inc, Raleigh, NC, USA), CD4 (YKIX302.9; Serotec Inc, Raleigh, NC, USA), and CD8 (YCATE55.9; Serotec Inc, Raleigh, NC, USA)], B-lymphocyte marker CD21 (CA2.1D6; Serotec Inc, Raleigh, NC, USA), and precursor cell marker CD34 (1H6; B-D Biosciences, San Jose, CA, USA) (Colorado state university, Fort Collins, CO, USA). After the exclusion of dead cells by propidium iodide staining, populations of lymphocyte were gated on the forward scatter vs. log side scatter plots (12). Neoplastic lymphocytes predominantly consisted of CD21+ cells with 66.73% of all nucleated cells in the blood sample and 31.71% of all nucleated cells in the bone marrow sample, respectively. Among lymphocytes, cells with CD21 expression were 87.52% in the blood, and 75.60% in the bone marrow, respectively. These results revealed a B-cell origin, which indicated B-cell CLL (Figures 2A,B). Expression of the remaining markers as mentioned above was unremarkable. Moreover, polymerase chain reaction for antigen receptor rearrangements (PARR) using blood samples was performed to determine clonality by detecting rearranged antigen receptor genes (Colorado state university, Fort Collins, CO, USA). DNA was extracted from blood, and the size of the antigen receptor hypervariable region was detected by PCR with amplification of immunoglobulin for B cells and T-cell receptor γ sequences for T cells (12). PARR results of the dog showed a positive reaction to immunoglobulin sequence with prominent bands while a negative reaction was shown to T-cell receptor γ sequence as no clonal bands, indicating B-cell clonality.

### Treatment and Outcome

Treatment was initiated with chlorambucil (0.2 mg/kg, PO, QOD; Excella GmbH, Germany) and prednisolone (30 mg/m^2^ surface area, PO, SID; Daesung, Korea) 28 days after the first admission (Table 1, Day 0). One week later, the chlorambucil dosage was reduced to 0.1 mg/kg (PO, QOD), and prednisolone dosage was reduced to 20 mg/m^2^ (PO, SID). After another week, the chlorambucil dosage was reduced to 0.1 mg/kg (PO, EOD), and prednisolone dosage was reduced to 10 mg/m^2^ (PO, EOD). Moreover, additional medication was prescribed, involving the administration of liver supplement medications (Zentonil; 0.1 T/kg, PO, divided; Vetoquinol, USA.) and ursodeoxycholic acid (10 mg/kg, PO, BID; Daewoong, Korea) for the control of elevated liver enzymes.

The dog's leukocyte and lymphocyte counts did not resolve remarkably after 3 weeks of chlorambucil and prednisolone chemotherapy (Figure 1C). Moreover, clinical signs including hyperthermia, tachypnea, and hepatosplenomegaly were not resolved. Therefore, imatinib (10 mg/kg, PO, SID; Novartis, Switzerland) was additionally administrated to the original therapy (Table 1, Day 22). Two weeks after combined treatment, the white blood cell (WBC) counts decreased from 35.46 × 10^9^/L to 21.53 × 10^9^/L, and the imatinib dosage was decreased from once daily to every other day to prevent myelosuppression. Moreover, hyperthermia was resolved, and the dog had normal body temperature (rectal temperature: 38.9°C). However, 9 weeks later, WBC counts increased to 30.4 × 10^9^/L (Table 1, Day 100). Therefore, the imatinib dosage was increased to once daily. After 2 weeks, WBC counts increased from 30.4 × 10^9^/L to 32.32 × 10^9^/L and mild anemia (HCT 36.7%; reference range, 37.3–61.7%) occurred as a side effect of myelosuppression. Considering deterioration of the side effect, the dosage of imatinib was changed from once daily to 2 days taking on and 1 day taking off in a 3-day cycle. Moreover, 18 days after additional administration of imatinib, test for c-kit mutation in the blood sample was performed to check mutations identified in the c-kit gene (Colorado state university, Fort Collins, CO, USA). There was no internal tandem duplication in c-kit exons 8 and 11, indicating no c-kit mutation in the dog. After 2 weeks, WBC counts dramatically decreased to 16.86 × 10^9^/L, and anemia was resolved (Table 1, Day 127). WBC and lymphocyte count of this dog was well-controlled with the current treatment regimen. On day 386, WBC counts were within the normal range (15.41 × 10^9^/L; reference range, 5.05–16.76 × 10^9^/L), and lymphocyte counts were also within the normal range (3.2 × 10^9^/L; reference range, 1.05–5.1 × 10^9^/L; Figure 1D).

Approximately 1 year after starting treatment, flowcytometry was re-assessed for evaluating the current treatment response (Figure 2C). The number of cells expressing the CD21 molecule decreased from 24,678/μL at the first admission date to 704/μL (reference range, 85–350) showing a highly favorable response to medication (Figure 2D). The dog responded well to therapy with chlorambucil, prednisolone, and imatinib for 2 years and remained clinically normal.

## Discussion

The median survival time for dogs with CLL was reported as ~1 year among 17 dogs with CLL treated with prednisone, chlorambucil, and vincristine (13), and another report showed that 17 dogs with B-cell CLL had a median overall survival time of 480 days (9). Poor prognostic factors of CLL include the large B-cell type, young age <8 years, development of Richter's syndrome, paraneoplastic syndromes including immune-mediated hemolytic anemia and pure red cell aplasia, and lymphocytosis of more than 30,000 lymphocytes/μL in the CD8+ phenotype (1, 9, 12, 14). The dog in this case had poor prognostic factors, including age <8 years, but the clinical response to the combination therapy of chlorambucil, prednisolone, and imatinib was excellent over a 2-year follow-up period without severe adverse effects.

Imatinib, a small-molecule tyrosine kinase inhibitor, was originally developed for the treatment of human chronic myeloid leukemia and has been used off-label in dogs and cats with neoplasms such as mast cell tumors (15). Imatinib can be useful in dogs and cats with mutations in the tyrosine kinases because the therapeutic effect of imatinib is linked to the activation of KIT (15). It has been reported that imatinib can sensitize CLL cells to the cytotoxic effects of chlorambucil and can induce apoptosis in CLL cells *in vitro* (16). Imatinib inhibits c-ABL kinase activity resulting in a decrease in Rad51 phosphorylation, leading to a repair of chlorambucil-induced DNA damage (16). CLL patients are more likely to have increased c-ABL in humans (17, 18). Moreover, one human case study reported that imatinib was used effectively for the treatment of CLL and chronic myelogenous leukemia, showing decrease of peripheral blood CLL cells (19). Although no study in veterinary medicine has shown clinical response of CLL in dogs treated with imatinib, we hypothesized that imatinib would show similar efficacy in dogs in that clinical efficacy of imatinib has been revealed in humans with CLL and imatinib has an ability to induce cytotoxic effect of chlorambucil synergistically *in vivo*. Therefore, imatinib was added to the original therapy with prednisolone and chlorambucil in this case, and CLL was well-controlled, with normalization of WBC levels. Although the dog in this case had no internal tandem duplication in c-kit exons 8 and 11, indicating no c-kit mutation, the dog showed favorable outcome. Therefore, for dogs with CLL having a poor response to chlorambucil, imatinib could be additionally administrated regardless of c-kit mutation. Moreover, the treatment cost for chlorambucil in combination with imatinib is affordable in practice rather than systemic parenteral chemotherapy.

Flow cytometric analysis was performed twice in this dog to characterize lymphocytosis at the first visit and for evaluation of the multimodality therapy response 1 year later. The number of lymphocytes predominantly expressing the CD21 molecule was remarkably decreased to normal following 1 year of treatment.

In conclusion, this is the case report describing the clinical response of a dog with CLL treated with chlorambucil and prednisolone in combination with imatinib. To the best of the authors' knowledge, no study has shown clinical outcome of CLL in dogs treated with this multimodality medication. This case report indicates that this combination is well-tolerated and could have a favorable anticancer effect in canine CLL if the treatment dosage is properly adjusted in accordance with the WBC count.

## Data Availability Statement

The original contributions presented in the study are included in the article/supplementary material, further inquiries can be directed to the corresponding author/s.

## Ethics Statement

Ethical review and approval was not required for the animal study because informed consent was obtained from the present owner of the dog for publication of this case report and any accompanying images.

## Author Contributions

G-WL was involved in case analysis and was responsible for writing the manuscript. M-HK and J-HJ were involved in the draft preparation and case analysis. D-WS, W-BR, and H-SK were involved in case analysis and reviewing the manuscript. H-MP was involved in the coordination of the case and was responsible for interpretation of results. All authors contributed to the article and approved the submitted version.

## Conflict of Interest

The authors declare that the research was conducted in the absence of any commercial or financial relationships that could be construed as a potential conflict of interest.

## Publisher's Note

All claims expressed in this article are solely those of the authors and do not necessarily represent those of their affiliated organizations, or those of the publisher, the editors and the reviewers. Any product that may be evaluated in this article, or claim that may be made by its manufacturer, is not guaranteed or endorsed by the publisher.
